# Evaluation of adalimumab biosimilar candidate (HS016) in Chinese patients with active ankylosing spondylitis based on a health survey: sub-analysis of a phase 3 study

**DOI:** 10.1007/s10067-021-05943-w

**Published:** 2021-10-28

**Authors:** Jinmei Su, Mengtao Li, Lan He, Dongbao Zhao, Weiguo Wan, Yi Liu, Jianhua Xu, Jian Xu, Huaxiang Liu, Lindi Jiang, Huaxiang Wu, Xiaoxia Zuo, Cibo Huang, Xiumei Liu, Fen Li, Zhiyi Zhang, Xiangyuan Liu, Lingli Dong, Tianwang Li, Haiying Chen, Jingyang Li, Dongyi He, Xin Lu, Anbin Huang, Yi Tao, Yanyan Wang, Zhuoli Zhang, Wei Wei, Xiaofeng Li, Xiaofeng Zeng

**Affiliations:** 1 Department of Rheumatology, Peking Union Medical College Hospital, Peking Union Medical College and Chinese Academy of Medical Sciences, National Clinical Research Center for Immunologic Diseases, Ministry of Science and Technology, Key Laboratory of Rheumatology and Clinical Immunology, Ministry of Education, No. 1 Shuaifuyuan, Dongcheng District, Beijing, 100730 China; 2 Department of Rheumatology and Immunology, The First Affiliated Hospital of Xi’an Jiaotong University, Xi’an, China; 3 Department of Rheumatology, Changhai Hospital, Shanghai, China; 4 Department of Rheumatology, Huashan Hospital, Fudan University, Shanghai, China; 5 Department of Rheumatology, West China Hospital, Sichuan University, Chengdu, China; 6 Department of Rheumatology, The First Affiliated Hospital of Anhui Medical University, Hefei, China; 7 Department of Rheumatology, The First Affiliated Hospital of Kunming Medical University, Kunming, China; 8 Department of Rheumatology, Qilu Hospital of Shandong University, Jinan, China; 9 Department of Rheumatology, Zhongshan Hospital, Fudan University, Shanghai, China; 10 Department of Rheumatology, The Second Affiliated Hospital of Zhejiang University School of Medicine, Hangzhou, China; 11 Department of Rheumatology, Xiangya Hospital, Central South University, Changsha, China; 12 Department of Rheumatology, Beijing Hospital, Beijing, China; 13 Department of Rheumatology, The First Affiliated Hospital of Shanxi Medical University, Taiyuan, China; 14 Department of Rheumatology, The Second Xiangya Hospital of Central South University, Changsha, China; 15 Department of Rheumatology, The First Affiliated Hospital of Harbin Medical University, Harbin, China; 16 Department of Rheumatology, Peking University Third Hospital, Beijing, China; 17 Department of Rheumatology, Tongji Hospital, Tongji Medical College of Huazhong University of Science and Technology, Wuhan, China; 18 Department of Rheumatology, Guangdong Second Provincial General Hospital, Guangzhou, China; 19 Department of Rheumatology, The Third Hospital of Hebei Medical University, Shijiazhuang, China; 20 Department of Rheumatology, Zhuzhou Central Hospital, Zhuzhou, China; 21 Department of Rheumatology, Shanghai Guanghua Hospital of Integrated Traditional Chinese and Western Medicine, Shanghai, China; 22 Department of Rheumatology, China-Japan Friendship Hospital, Beijing, China; 23 Department of Rheumatology, Union Hospital, Tongji Medical College of Huazhong University of Science and Technology, Wuhan, China; 24 Department of Rheumatology, The Second Affiliated Hospital of Guangzhou Medical University, Guangzhou, China; 25 Department of Rheumatology, Jiangsu Province Hospital, Nanjing, China; 26 Department of Rheumatology, Peking University First Hospital, Beijing, China; 27 Department of Rheumatology, Tianjin Medical University General Hospital, Tianjin, China; 28 Department of Rheumatology, The Second Affiliated Hospital of Shanxi Medical University, Taiyuan, China

**Keywords:** Adalimumab, Ankylosing spondylitis, Biosimilar, HS016

## Abstract

**Objective:**

The equivalence of the biosimilar HS016 to adalimumab (Humira) for the treatment of active ankylosing spondylitis (AS) patients has been previously validated. The aim was to compare the efficacy of HS016 and adalimumab in stratified subgroups at different time points using Health Assessment Questionnaire for Spondyloarthropathies (HAQ-S) and short form 36 (SF-36) questionnaires.

**Methods:**

We carried out a multicenter, randomized, double-blind, parallel, positive control, phase 3 trial of patients with active AS. They were selected randomly to be subcutaneously administered 40 mg HS016 or adalimumab every 2 weeks for a total treatment period of 24 weeks in a 2:1 ratio. A health surveys were used to assess mental and physical improvements of patients as well as other factors.

**Results:**

HAQ-S revealed that changes in scores from baseline in both groups were time dependent until 14 weeks and that during the first 4 weeks of treatment the changes declined rapidly. The SF-36 health survey revealed that both HS016 and adalimumab produced rapid beneficial effects against AS during the first 2 weeks of therapy, which gradually declined between 2 and 12 weeks and flattened out after 12 weeks until 24 weeks.

**Conclusion:**

This trial demonstrated that both HS016 and adalimumab produced rapid improvements in symptoms during the first 2 weeks of treatment. These findings suggest that HS016 is an alternative economical treatment for Chinese AS patients producing a rapid amelioration of symptoms, aiding them to recover their lifestyle satisfaction.

**Trial registration:**

http://www.chictr.org.cn/enindex.aspx, ChiCTR1900022520, retrospectively registered.

**Table:** 

Key points
*• HS016 and adalimumab produced rapid AS symptom improvements during the first 2 weeks followed by a slowdown of improvements until week 4 with afterwards few improvements evaluated by HAQ-S*
*• The improvements according to the short form of the 36 (SF-36) questionnaires revealed similar trends as for HAQ-S*
*• There was no significant difference in HAQ-S and SF-36 scores between HS016 and adalimumab*

**Supplementary Information:**

The online version contains supplementary material available at 10.1007/s10067-021-05943-w.

## Introduction

Ankylosing spondylitis (AS) is an inflammatory disease affecting spinal joints, causing back pain and stiffness. In advanced cases, it can lead to spinal deformity [1] and major disability and produces significant socioeconomic consequences [2]. As part of the Medical Outcomes Study (MOS), the RAND cooperation developed a 36-item short-form health survey (SF-36), which was primarily designed to be used in the clinic but also for research, evaluation of health policies, and to survey the general population [3]. The questionnaire addresses 36 issues grouped into 8 domains, namely physical function, role physical, bodily pain, general health, vitality, social function, and the role emotional and mental health [4]. Patient self-reporting provides data which are widely utilized to monitor and assess the aftermath of adult patient care [5, 6]. In addition, Health Assessment Questionnaire for Spondyloarthropathies (HAQ-S) is often used to evaluate of the functions and health status of AS patients [7]. The original HAQ came in two versions [8]. One contained 5 dimensions of health outcomes and the other one was a short HAQ, which included the HAQ disability index (HAQ-DI) and the visual analog scales (VAS) of pain and stiffness [9]. Liu et al. [10] reported (2017) that the Chinese version of HAQ-S was suitable to assess Chinese-speaking AS patients, because the Chinese version correlated well with the Bath AS Functional Index (BASFI), and only moderately with the Bath AS Disease Activity Index (BASDAI) and Bath AS Metrology Index (BASMI).

Our previous results demonstrated that HS016 produced similar effects to adalimumab in terms of its safety and efficacy during a treatment period of 24 weeks, measured at baseline, week 12 and week 24. There were no significant differences of Assessment of SpondyloArthritis International Society (ASAS)20, ASAS40, ASAS5/6 scores, BASDAI improvements and severity of morning stiffness, treatment-emergent adverse events, and pharmacokinetics as well as positive neutralizing antibody (NAb) developments between the groups [11]. In the current study, the therapeutic efficacies of the two drugs were compared with HAQ-S and health survey (SF-36) outcomes monitored every 2 weeks from baseline until week 24 in order to find differences during the time course of treatments for the patients’ quality of life.

## Materials and methods

### Design of the study

Data for this study were derived from a phase 3 clinical trial carried out in China on patients with active AS. A total of 603 active AS patients were enrolled and randomly assigned to the test group or control group at a ratio of 2:1.

All patients subcutaneously received 12 injections of 40 mg/0.8 mL of HS016 or adalimumab every 2 weeks for 24 weeks. The treatment period lasted for 28 weeks and consisted of 2 weeks screening, 24 weeks therapy, and 2 weeks follow-up. Every 2 weeks, patients attended the research center and were injected with the appropriate study drugs. Health surveys including HAQ-S and SF-36 were evaluated at each visit.

The study was registered with the Chinese Clinical Trial Registry (no. ChiCTR1900022520) and carried out by strictly following the guidelines of the Good Clinical Practice and Provisions for Drug Registration of the National Medical Products Administration (NMPA). An ethics committee at every participating center granted approval of the study protocols and reviewed all amendments. All patients provided signed informed consent before they were enrolled in the trial.

### Randomization

An independent contract research organization produced a random table of items. The item randomization table contained the treatment group and random numbers, which was input to our central random system (IWRS). After confirming that patients met the criteria for inclusion in the study, each test center conducted patient randomization using IWRS and assigned each one a random number. The center administered the appropriate drug to a patient according to their random number. The randomized double-blind design used in this study ensured that the investigator, relevant researchers, and patients were blinded to the test group.

### Patients

The target population were active AS patients who voluntarily signed informed consent and were able to comply with the scheme and had the ability to carry out relevant procedures.

### Inclusion and exclusion criteria

We enrolled patients with AS who met the modified New York criteria 1984 [12] with or without peripheral joint involvement. We also included those AS patients with still active peripheral joint involvement, which were treated with disease-modifying antirheumatic drugs (DMARDs). Patients had to meet two or more of the following conditions: (1) A BASDAI score ≥ 4; (2) In VAS assessment, total back pain was ≥ 4 cm; and (3) Morning stiffness time ≥ 1 h, previously used ≥ 1 non-steroidal anti-inflammatory drugs (NSAIDs) or ≥ 1 DMARDs for at least 4 weeks, where the drugs had been ineffective or patients could not tolerate one additional NSAID. According to Chinese guidelines, these patients were suitable for treatment with TNF-α antagonists [13].

Patients who exhibited total spinal rigidity or had spinal surgery or joint surgery 24 weeks before the initiation of the trial, or who had been treated with TNFα antagonists 12 weeks prior to randomization, were excluded. Additional information on the inclusion and exclusion criteria has been reported elsewhere [11].

### Endpoints

Changes of various indexes in the health surveys (Chinese versions of HAQ-S [10] and SF-36 [14–16]) at different time points during the whole treatment period were monitored. Data at each time point were only collected from patients who actually received the treatments. In the case of discontinuation, further HAQ-S and SF-36 data were not evaluated for these cases. The questionnaire of SF-36 includes 8 domains including body disability and stiffness, physical function, role physical, bodily pain, general health, vitality, social function, and role emotional and mental health. In addition, mental health composite score (MCS) and the physical health composite score (PCS) are also assessed according to the following Eqs. (1) and (2). Because MCS and PCS correlate with SF-36 scales, they are weighed by the appropriate coefficients of physical or mental factor before aggregation to form the two summary scores. Norm-based scoring with z-score transformation ((observed score-population mean)/population standard deviation) and standardization of the population mean and standard deviation (SD) to 50–10, respectively, are recommended for easier interpretation [17].

1 $$\text{SF-36 PCS}{=}\sum \left(\text{z score of each scale}\times \text{respective physical factor coefficient}\right)\times 10+50$$

2 $$\text{SF-36 MCS}=\sum \left(\text{z score of each scale}\times \text{respective mental factor coefficient}\right)\times 10+50$$

The instrument of short form HAQ includes the HAQ-DI and the VAS of pain and stiffness. The HAQ-DI comprised of 8 subdivisions (dressing and grooming, arising, eating, walking, hygiene, reaching, gripping, common daily activities) with each subscale involving 2–3 activities. The score ranges for each measurement were 0–3, with a high score indicating poorer functions. Eight subscale scores plus the VAS of pain and stiffness were averaged to produce a mean of the HAQ-S score ranges [9, 10].

### Statistical analysis

SAS (ver. 9.2) was used for all data analyses. Quantitative indexes are presented as the mean ± SD or the median (min, max). Qualitative or grade indicators are expressed as the number of cases and percentages. The full analysis set (FAS) included all patients randomly assigned to receive at least one treatment after randomization according to the intention-to-treat (ITT) principle. The FAS was used for baseline and effectiveness analyses.

## Results

### Demographic information of enrolled patients

Six hundred and forty-nine patients that met the trial criteria were enrolled. A total of 648 (99.8%) patients received treatments, of which 570 (87.8%) completed the study (362/416 (87.0%) in the HS016 group and 208/232 (89.7%) in the adalimumab group) (Supplementary Fig. 1 and Supplementary Table 1). HAQ-S and SF-36 scores were both derived from 648 patients.

The majority in the HS016 and adalimumab groups were mostly males (86.3% and 87.9%, respectively) under 40 years of age (85.3% and 78.4%, respectively). Their duration of AS were 6.37 ± 5.24 and 6.49 ± 5.73 years, respectively. In addition, there were no significant differences in erythrocyte sedimentation rate (ESR) and C-reactive protein (CRP) between the 2 groups at the baseline stage. Overall, the demographic characteristics of enrolled patients in the two groups were broadly comparable (Table 1).

**Table 1:** Baseline characteristics of patients

Index	HS016 (*n* = 416)	Adalimumab (*n* = 232)	*P*-value
Age (year)	31.46 ± 7.84	32.11 ± 8.88	0.333
Age stratification, *n* (%)			0.026
< 40 years	355 (85.3)	182 (78.5)	
≥ 40 years	61 (14.7)	50 (21.6)	
BMI (kg/ m^2^)	23.29 ± 2.38	23.25 ± 2.50	0.843
Gender, *n* (%)			0.555
Male	359 (86.3)	204 (87.9)	
Female	57 (13.7)	28 (12.1)	
Course of disease (year)	6.37 ± 5.24	6.49 ± 5.73	0.928
ESR (mm/h)	29.38 ± 23.82	31.24 ± 22.35	0.331
CRP (mg/L)	29.67 ± 33.78	31.39 ± 31.53	0.523
ASDAS-CRP	3.95 ± 0.84	4.04 ± 0.88	0.196

Data is presented as mean ± SD or numbers with percentage (%). *CRP*, C-reactive protein; *ESR*, erythrocyte sedimentation rate; *ASDAS*, Ankylosing Spondylitis Disease Activity Score

### Improvement of HAQ-S scores

Health survey HAQ-S scores were evaluated for AS patients during the 24 weeks treatment period. At the baseline stage, there were no significant differences among 3 indexes, namely disability including eight subscales (dressing and grooming, arising, eating, walking, hygiene, reaching, gripping, common daily activities), between the HS016 and adalimumab groups (Table 2). From baseline to treatment for 2 weeks, HAQ-S scores were reduced to 0.42 ± 0.37 and 0.45 ± 0.34 from 0.57 ± 0.40 and 0.61 ± 0.41 in the HS016 and adalimumab groups, respectively. It was clear that there was a rapid improvement of 26.3% in the HS016-treated patients and 26.2% in the adalimumab-treated patients after 2 weeks of therapy. After 4 weeks, the improvement was 38.6% from baseline for HS016 and 36.1% for adalimumab. When treatment lasted for 6 weeks, the improvement of health status was changed to gradually increased and maintenance. After 24 weeks treatment, the HAQ-S score was improved to 54.4% of baseline in HS016 group and 54.1% of baseline in the adalimumab group.

**Table 2:** Comparison of HAQ-S scores evaluated at different treatment times from baseline between the two groups

	HAQ-S		*P*-value
	HS016 (*n* = 416)	Adalimumab (*n* = 232)	
Baseline	0.57 ± 0.40	0.61 ± 0.41	0.287
Week 2	0.42 ± 0.37	0.45 ± 0.34	0.398
Week 4	0.36 ± 0.34	0.39 ± 0.32	0.292
Week 6	0.33 ± 0.33	0.36 ± 0.32	0.288
Week 8	0.32 ± 0.32	0.34 ± 0.30	0.415
Week 10	0.30 ± 0.31	0.32 ± 0.31	0.422
Week 12	0.30 ± 0.31	0.31 ± 0.30	0.636
Week 14	0.28 ± 0.31	0.30 ± 0.30	0.533
Week 16	0.29 ± 0.31	0.29 ± 0.29	0.756
Week 18	0.28 ± 0.31	0.29 ± 0.30	0.763
Week 20	0.27 ± 0.30	0.29 ± 0.29	0.388
Week 22	0.26 ± 0.30	0.28 ± 0.28	0.459
Week 24	0.26 ± 0.30	0.28 ± 0.29	0.459

All data is presented as mean ± SD

Taking disability as one function of HAQ-S (Table 3), at the baseline, AS patients who found normal activity difficulty (including with much difficulty activity and unable to do) included 71 patients in the HS016 group and 56 in the adalimumab group. After 24 weeks treatment, only 27 patients treated with HS016 were left in this status, and 16 patients treated with adalimumab had significant disability. This means that either HS016 or adalimumab treatment could improve similar abilities of AS patients.

**Table 3:** The numbers of the patients in disability of HAQ-S from baseline in the two groups

	Complete activities		Without any difficulty		With some difficulty activity		With much difficulty activity		Unable to do	
	HS016 (*n* = 416)	Adalimumab (*n* = 232)	HS016 (*n* = 416)	Adalimumab (*n* = 232)	HS016 (*n* = 416)	Adalimumab (*n* = 232)	HS016 (*n* = 416)	Adalimumab (*n* = 232)	HS016 (*n* = 416)	Adalimumab (*n* = 232)
Baseline	33 (7.9)	19 (8.2)	121 (29.1)	59 (25.4)	191 (45.9)	98 (42.2)	66 (15.9)	54 (23.3)	5 (1.2)	2 (0.9)
Week 2	48 (11.5)	25 (10.8)	141 (33.9)	76 (32.8)	172 (41.4)	106 (45.7)	50 (12.0)	22 (9.5)	5 (1.2)	3 (1.3)
Week 4	54 (13.0)	35 (15.1)	152 (36.5)	70 (30.2)	173 (41.6)	95 (41.0)	35 (8.4)	32 (13.8)	2 (0.5)	0 (0.0)
Week 6	60 (14.4)	34 (14.7)	149 (35.8)	74 (31.9)	164 (39.4)	96 (41.4)	40 (9.6)	28 (12.1)	3 (0.7)	0 (0.0)
Week 8	57 (13.7)	36 (15.5)	157 (37.7)	68 (29.3)	162 (38.9)	101 (43.5)	38 (9.1)	26 (11.2)	2 (0.5)	1 (0.4)
Week 10	64 (15.4)	40 (17.2)	156 (37.5)	76 (32.8)	161 (38.7)	91 (39.2)	34 (8.2)	24 (10.3)	1 (0.2)	1 (0.4)
Week 12	55 (13.2)	42 (18.1)	165 (39.7)	67 (28.9)	160 (38.5)	105 (45.3)	35 (8.4)	17 (7.3)	1 (0.2)	1 (0.4)
Week 14	61 (14.7)	36 (15.5)	151 (36.3)	67 (28.9)	168 (40.4)	110 (47.4)	35 (8.4)	18 (7.8)	1 (0.2)	1 (0.4)
Week 16	61 (14.7)	40 (17.2)	155 (37.3)	71 (30.6)	165 (39.7)	106 (45.7)	34 (8.2)	13 (5.6)	1 (0.2)	2 (0.9)
Week 18	61 (14.7)	36 (15.5)	155 (37.3)	67 (28.9)	168 (40.4)	115 (49.6)	31 (7.5)	12 (5.2)	1 (0.2)	2 (0.9)
Week 20	68 (16.4)	38 (16.4)	154 (37.0)	63 (27.2)	163 (39.2)	113 (48.7)	30 (7.2)	16 (6.9)	1 (0.2)	2 (0.9)
Week 22	66 (15.9)	41 (17.7)	152 (36.5)	62 (26.7)	166 (39.9)	112 (48.3)	31 (7.5)	16 (6.9)	1 (0.2)	1 (0.43)
Week 24	75 (18.0)	40 (17.2)	143 (34.4)	65 (28.0)	171 (41.1)	111 (47.8)	26 (6.3)	14 (6.0)	1 (0.2)	2 (0.86)

Data is presented as numbers with percentage

**Fig. 1:**
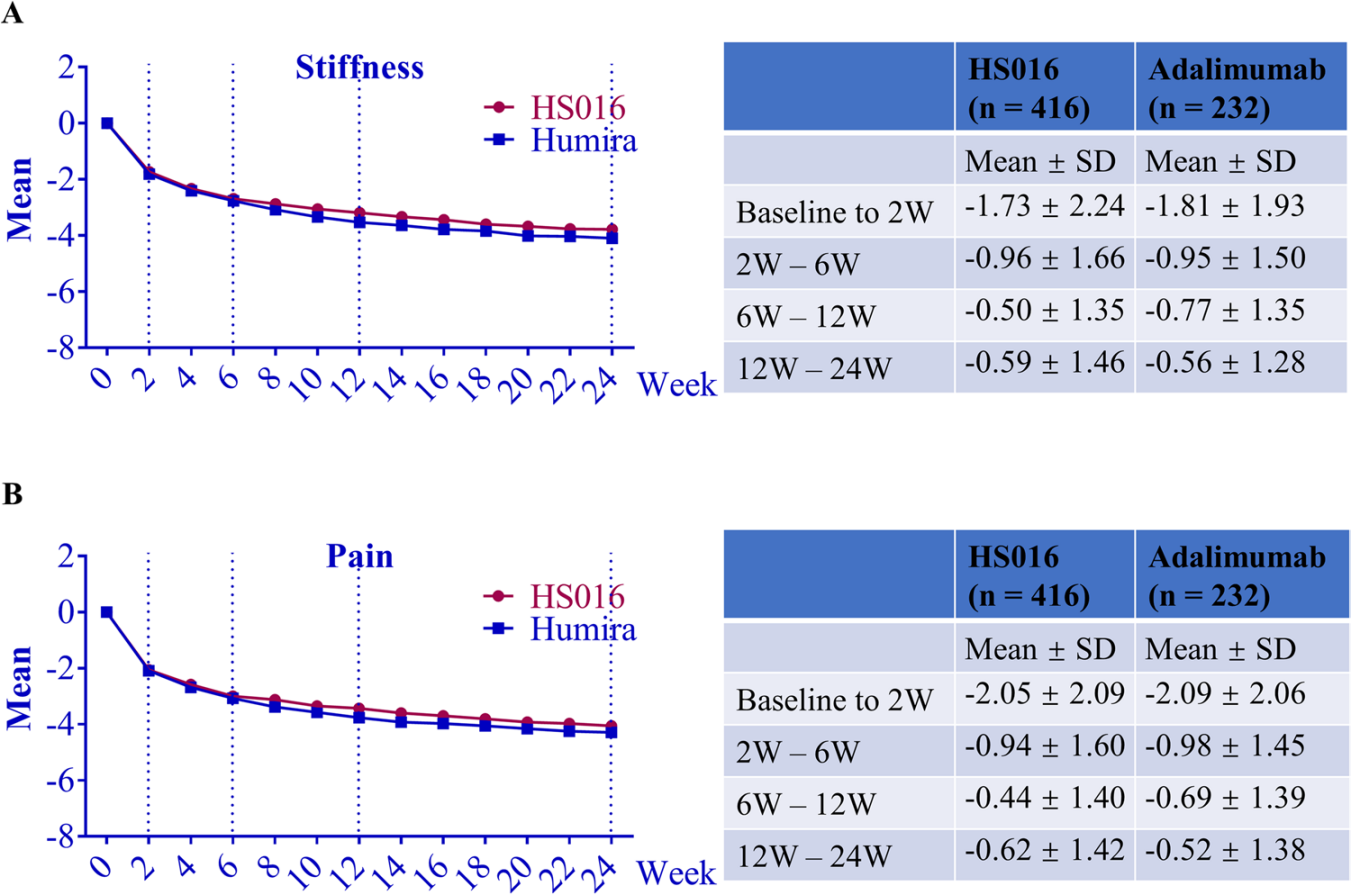
Improvement of the HAQ-S during 2 weeks. The changing scores of stiffness (**A**) and pain (**B**) were plotted on the left side. The data from baseline to 2 weeks, 2–12 weeks, and 12–24 weeks are summarized in the corresponding tables

We also analyzed the changing rate of stiffness and pain scores over a 24-week treatment period (Fig. 1). During the first 2 weeks of treatment, the changing rate of stiffness in the HS016 and adalimumab groups (Fig. 1A and Supplementary Table 2) was 1.73 ± 2.24 and − 1.81 ± 1.93, respectively. During weeks 2–6, the changing rates decreased to − 0.96 ± 1.66 and − 0.95 ± 1.50, respectively. During the 6–12 week and 12–24 weeks treatment periods, it almost reached a steady state. Very similar results were found for the pain indicators (Fig. 1B and Supplementary Table 3) and all patients achieved about 85.0% improvements in stiffness and pain scores at 12 weeks (Fig. 1A and 1B).

### Improvement of SF-36 indexes

**Fig. 2:**
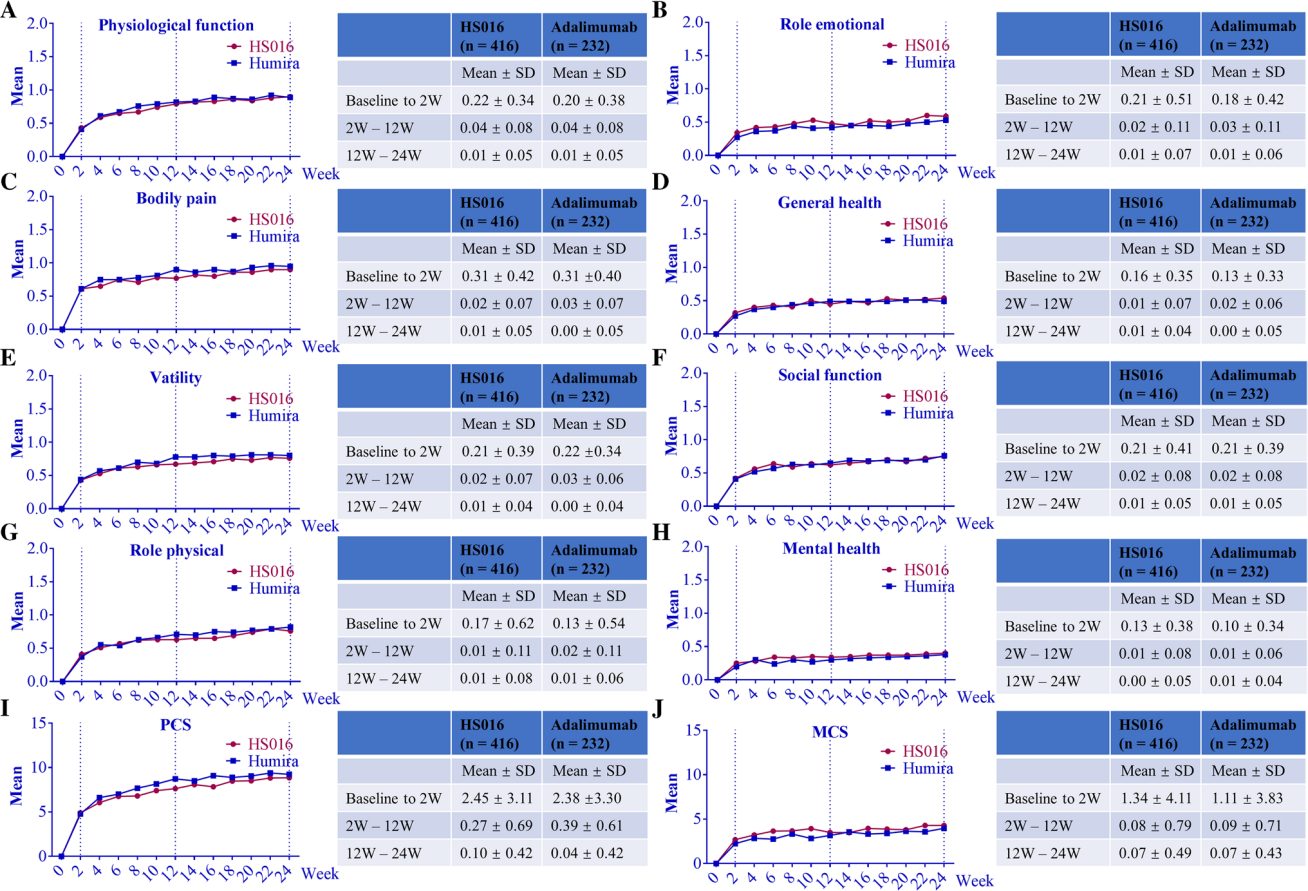
Improvement of the SF-36 health survey. The changing scores of physiological fuction (**A**), role emotional (**B**), bodily pain (**C**), general health (**D**), vatility (**E**), social function (**F**), role physical (**G**), mental health (**H**), PCS (**I**) and MSC (**J**) were plotted on the left side. The changing rates from baseline to 2 weeks, 2–12 weeks, and 12–24 weeks are summarized in the corresponding tables. MCS, mental health composite score; PCS, physical health composite score

Health survey SF-36 was also employed to record the improvement of AS patients during the 24-week treatment period. At baseline, there were no obvious differences among 10 indexes of the SF-36 between the two groups (Table 4). Among the 10 indexes, the improvements compared to baseline at different time points were analyzed. Overall, no significant differences between the two groups with regard to these indexes were detected, which indicated that these two drugs had an equal effect on AS. Taking physiological function as an example (Fig. 2A), after 24 weeks of treatment, the changing rates from baseline in physiological function increased from 0.43 (2 weeks) to 0.90 (24 weeks) in the HS016 group and from 0.41 (2 weeks) to 0.89 (24 weeks) in the adalimumab group. Other indicators showed similar results (see details in supplementary materials). We also analyzed the rate of change over the 24-week treatment period (Fig. 2). In the first 2 weeks of treatment, the speed of changing rates of physical function in the HS016 and adalimumab groups (Fig. 2A) was 0.22 ± 0.34 and 0.20 ± 0.38, respectively. During 2–12 weeks, the changing rate speeds decreased to 0.04 ± 0.08 in both groups. During the 12–24 weeks treatment, it almost reached a steady state (decreased to 0.01 ± 0.05 of the changing rate). Very similar results were found for the other 9 indicators.

**Table 4:** Scores of health survey (SF-36) at the baseline stage

	SF-36		*P*-value
	HS016 (*n* = 416)	Adalimumab (*n* = 232)	
Physical function	− 1.38 ± 0.95	− 1.46 ± 1.00	0.353
Role physical	− 1.90 ± 0.82	− 2.01 ± 0.76	0.088
Bodily pain	− 1.61 ± 0.80	− 1.69 ± 0.79	0.188
General health	− 2.14 ± 0.93	− 2.15 ± 0.97	0.858
Vitality	− 1.01 ± 0.85	− 1.09 ± 0.84	0.239
Social function	− 1.59 ± 0.96	− 1.64 ± 0.94	0.480
Role emotional	− 1.58 ± 1.15	− 1.54 ± 1.18	0.715
Mental health	− 0.90 ± 0.98	− 0.91 ± 1.00	0.967
PCS	31.87 ± 7.55	30.78 ± 7.84	0.082
MCS	39.55 ± 9.67	39.74 ± 10.36	0.816

All data is presented as mean ± SD. *MCS*, mental health composite score; *PCS*, physical health composite score

## Discussion

The aim of this sub-analysis investigation was to determine the efficacy of HS016 based on HAQ-S and SF-36 at 2-week time points compared to the reference drug adalimumab during 24 weeks of treatment in Chinese AS patients. AS is an insidious inflammatory condition that affects relatively young people generally < 40 years old [18]. At the baseline, AS patients exhibit the most difficulty in carrying out regular activity (71 (17.1%) in HS016-treated patients and 56 (24.1%) in the adalimumab group). After 24 weeks treatment, only 27 (6.5%) patients treated with HS016 were in this status, and 16 (6.9%) of patients in the adalimumab group. The HAQ-S indicator improved by 36.8% from baseline for HS016 and 42.6% for adalimumab after 4 weeks treatment. After 24 weeks treatment, the HAQ-S score was improved to 54.4% of baseline in HS016-treated patients and 54.1% patients who received adalimumab therapy, which is in the same range as in a previous study which included efficacies of adalimumab, etanercept, and infliximab treatments of ankylosing spondylitis with median HAQ improvement of 57.7% [19], which proved that the efficacy of the study and control drug on health survey results were the same and the highest effect could be detected during the first 4 weeks.

For the SF-36 scores, the rate of clinical change started high at 2 weeks, and then gradually declined from 2–12 weeks and finally became stable from 12–24 weeks. The mean overall differences of mean PCS and MCA scores from baseline to week 24 were 8.85 and 4.29 for HS016 as well as 9.24 and 3.97 for adalimumab, which is similar with the range of 6.9–7.3 for PCS and 2.7–3.7 for MCA scores reported in previous studies of adalimumab treatments of AS [20, 21]

SF-36 has been extensively employed to assess the health-related quality of life (HRQL) in patients with developing diseases. A study involving 210 AS patients employed SF-36 to evaluate life quality and showed that individuals with AS had significantly lower life quality compared to disease-free controls. It is noteworthy that PCS was affected more in comparison with MCS in both genders [5]. Comparisons of HRQL scores in individuals with rheumatoid or psoriatic arthritis and AS revealed that chronic inflammatory rheumatic disease had a clear detrimental effect on HRQL in both sexes and age groups and that physical activity was impaired more than mental and social abilities [22]. Therefore, except for HAQ-S, SF-36 is also a useful and easy tool to employ to assess the improvement of disease during treatment.

Although HAQ-S and SF-36 are two good tools for the evaluation of the disability and health status of patients with AS, the Bath questionnaires [23] and ASQoL questionnaires [24] were also useful self-reported functional instruments for AS. Moreover, the Chinese version of HAQ-S was well correlated with the BASFI and moderately with BASDAI and BASMI. In the present study, both HAQ-S and SF-36 questionnaires were useful for the evaluation of the disease status of AS patients on various levels (activity, functional capacity, radiological findings, metrological measures, global status, and quality of life).

The only significant difference in the baseline characteristics of the 2 patient groups was that the mean age of the HS016 group patients was younger than in the adalimumab-treated patients group. A previous study proposed a model combining age with several other such as baseline CRP levels as a good predictor of the response to anti-TNF therapy [25]. Since other factors including CRP serum concentrations were not different at baseline, the somewhat younger age of the HS016 patients had no effect on differences in the perception of pain, HAQ-S, SF-36, and stiffness scores throughout the study period.

Limitations of the present study were the small sample size and the short observation time.

In conclusion, the equivalent efficacy of HS016 to adalimumab was further validated based on healthy surveys HAQ-S and SF-36. The disease improvement at each time point was evaluated and demonstrated that both HS016 and adalimumab produced rapid effects against AS during the first 2 weeks of treatment and a gradual improvement between 2 and 12 week before flattening out after 12 weeks.

## Supplementary Information

Below is the link to the electronic supplementary material.

Supplementary file1 (DOCX 302 KB)

## Data Availability

The datasets used and/or analyzed during the current study are available from the corresponding author on reasonable request.
