# Supplementary material for: Evaluation of adalimumab biosimilar candidate (HS016) in Chinese patients with active ankylosing spondylitis based on a health survey: sub-analysis of a phase 3 study
Source: Clin Rheumatol. 2021 Oct 28;41(3):731–9. doi: 10.1007/s10067-021-05943-w (PMC8873115; doi:10.1007/s10067-021-05943-w)
Supplement: Supplementary file 1 — Supplementary file1 (DOCX 302 KB) [file 10067_2021_5943_MOESM1_ESM.docx]

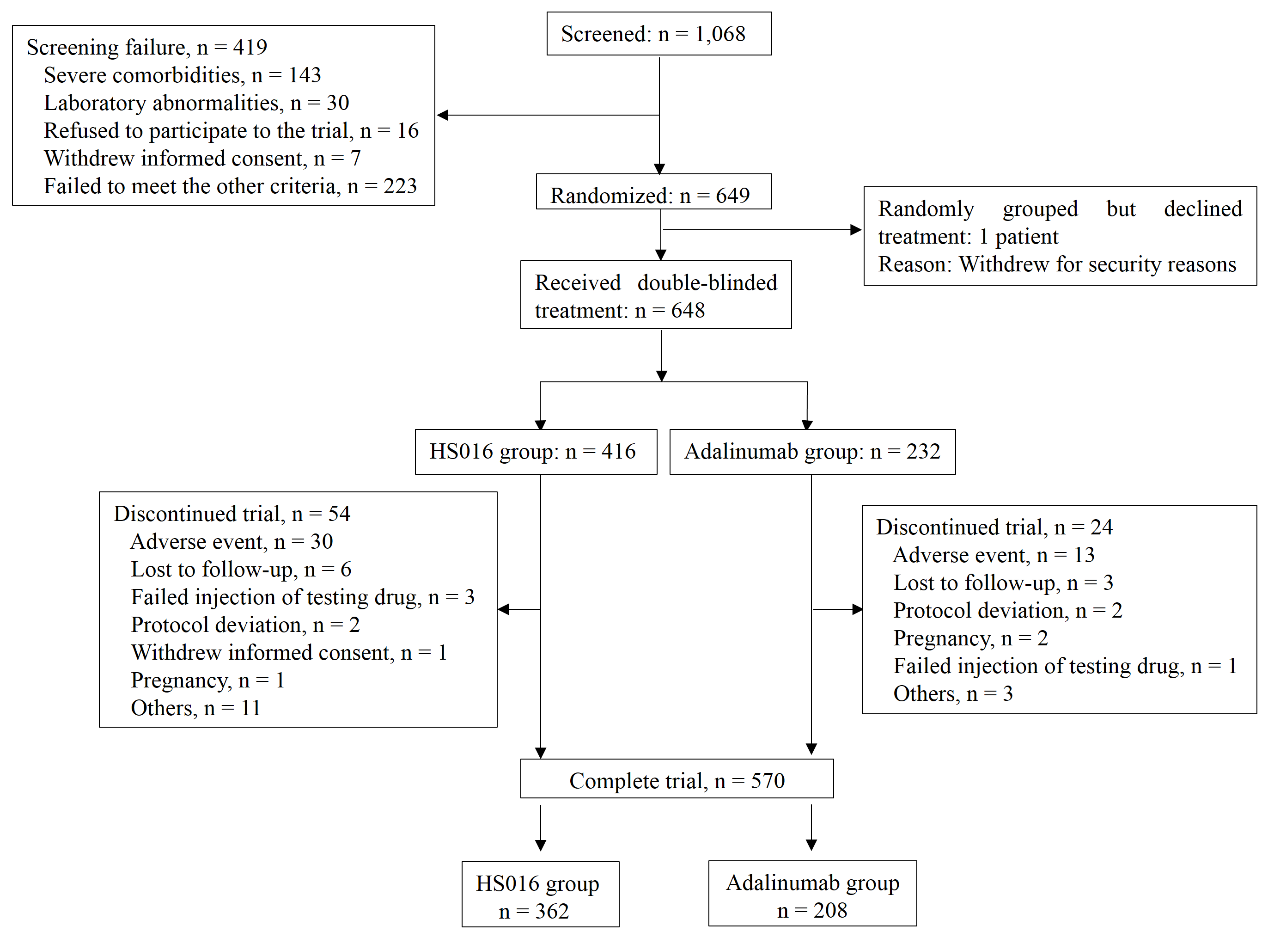


**Supplementary Figure 1. Flowchart of the study**

**Supplementary Table 1. Time points of discontinuations**

| **Visiting times** | **Groups** | | |
| --- | --- | --- | --- |
|  | **HS016** | **Humira** | **Total** |
| Baseline | 3 | 3 | 6 |
| Week 2 | 7 | 1 | 8 |
| Week 4 | 4 | 3 | 7 |
| Week 6 | 4 | 0 | 4 |
| Week 8 | 4 | 2 | 6 |
| Week 10 | 5 | 1 | 6 |
| Week 12 | 14 | 8 | 22 |
| Week 14 | 6 | 2 | 8 |
| Week 16 | 3 | 1 | 4 |
| Week 18 | 0 | 1 | 1 |
| Week 20 | 1 | 2 | 3 |
| Week 22 | 0 | 0 | 0 |
| Week 24 | 3 | 0 | 3 |
| Total | 54 | 24 | 76 |

**Supplementary Table 2.** **Changes** **in stiffness scores from baseline in the two groups**

|  | **Stiffness scores** | | ***P-*value** |
| --- | --- | --- | --- |
|  | **HS016 (n = 416)** | **Adalimumab (n = 232)** |  |
| Baseline | 6.24 ± 2.08 | 6.42 ± 1.95 | 0.281 |
| Week 2 | 4.50 ± 2.44 | 4.61 ± 2.28 | 0.602 |
| Week 2-baseline | -1.73 ± 2.24 | -1.81 ± 1.93 | 0.654 |
| Week 4 | 3.91 ± 2.52 | 4.02 ± 2.19 | 0.597 |
| Week 4-baseline | -2.33 ± 2.34 | -2.40 ± 2.11 | 0.682 |
| Week 6 | 3.55 ± 2.52 | 3.65 ± 2.23 | 0.598 |
| Week 6-baseline | -2.69 ± 2.39 | -2.76 ± 2.25 | 0.694 |
| Week 8 | 3.36 ± 2.46 | 3.34 ± 2.21 | 0.906 |
| Week 8-baseline | -2.87 ± 2.35 | -3.08 ± 2.32 | 0.289 |
| Week 10 | 3.19 ± 2.38 | 3.08 ± 2.22 | 0.569 |
| Week 10-baseline | -3.05 ± 2.34 | -3.34 ± 2.38 | 0.135 |
| Week 12 | 3.05 ± 2.35 | 2.88 ± 2.16 | 0.382 |
| Week 12-baseline | -3.19 ± 2.37 | -3.53 ± 2.31 | 0.075 |
| Week 14 | 2.90 ± 2.40 | 2.77 ± 2.19 | 0.498 |
| Week 14-baseline | -3.33 ± 2.49 | -3.64 ± 2.35 | 0.123 |
| Week 16 | 2.80 ± 2.32 | 2.64 ± 2.14 | 0.383 |
| Week 16-baseline | -3.44 ± 2.41 | -3.78 ± 2.37 | 0.082 |
| Week 18 | 2.63 ± 2.25 | 2.58 ± 2.14 | 0.754 |
| Week 18-baseline | -3.60 ± 2.43 | -3.84 ± 2.34 | 0.229 |
| Week 20 | 2.57 ± 2.27 | 2.41 ± 2.08 | 0.383 |
| Week 20-baseline | -3.67 ± 2.47 | -4.01 ± 2.37 | 0.091 |
| Week 22 | 2.48 ± 2.19 | 2.39 ± 2.03 | 0.613 |
| Week 22-baseline | -3.76 ± 2.46 | -4.03 ± 2.37 | 0.177 |
| Week 24 | 2.46 ± 2.25 | 2.32 ± 2.02 | 0.426 |
| Week 24-baseline | -3.78 ± 2.53 | -4.10 ± 2.40 | 0.114 |

Note. All data is presented as mean ± SD.

**Supplementary Table 3. Changes in pain scores from baseline in the two groups**

|  | **Pain scores** | | ***P-*value** |
| --- | --- | --- | --- |
|  | **HS016 (n = 416)** | **Adalimumab (n = 232)** |  |
| Baseline | 6.68 ± 1.66 | 6.86 ± 1.73 | 0.199 |
| Week 2 | 4.63 ± 2.28 | 4.77 ± 2.16 | 0.430 |
| Week 2-baseline | -2.05 ± 2.09 | -2.09 ± 2.06 | 0.846 |
| Week 4 | 4.10 ± 2.32 | 4.18 ± 2.11 | 0.645 |
| Week 4-baseline | -2.58 ± 2.18 | -2.68 ± 2.18 | 0.603 |
| Week 6 | 3.68 ± 2.33 | 3.79 ± 2.14 | 0.575 |
| Week 6-baseline | -3.00 ± 2.26 | -3.07 ± 2.25 | 0.690 |
| Week 8 | 3.56 ± 2.31 | 3.48 ± 2.18 | 0.659 |
| Week 8-baseline | -3.12 ± 2.26 | -3.38 ± 2.37 | 0.168 |
| Week 10 | 3.33 ± 2.25 | 3.29 ± 2.12 | 0.809 |
| Week 10-baseline | -3.35 ± 2.28 | -3.57 ± 2.28 | 0.237 |
| Week 12 | 3.25 ± 2.25 | 3.09 ± 2.14 | 0.400 |
| Week 12-baseline | -3.43 ± 2.31 | -3.76 ± 2.27 | 0.079 |
| Week 14 | 3.08 ± 2.23 | 2.93 ± 2.09 | 0.419 |
| Week 14-baseline | -3.60 ± 2.34 | -3.92 ± 2.28 | 0.090 |
| Week 16 | 2.98 ± 2.22 | 2.88 ± 2.11 | 0.600 |
| Week 16-baseline | -3.70 ± 2.32 | -3.97 ± 2.31 | 0.153 |
| Week 18 | 2.88 ± 2.17 | 2.81 ± 2.12 | 0.678 |
| Week 18-baseline | -3.80 ± 2.31 | -4.05 ± 2.36 | 0.188 |
| Week 20 | 2.76 ± 2.13 | 2.71 ± 2.06 | 0.747 |
| Week 20-baseline | -3.92 ± 2.29 | -4.15 ± 2.32 | 0.216 |
| Week 22 | 2.71 ± 2.12 | 2.61 ± 1.96 | 0.533 |
| Week 22-baseline | -3.97 ± 2.29 | -4.25 ± 2.28 | 0.130 |
| Week 24 | 2.63 ± 2.17 | 2.57 ± 1.95 | 0.709 |
| Week 24-baseline | -4.05 ± 2.33 | -4.29 ± 2.24 | 0.200 |

Note. All data is presented as mean ± SD.
